# Exploring the relationship between super-leadership, self-leadership, and exercise commitment in university Taekwondo teams

**DOI:** 10.3389/fpsyg.2024.1323503

**Published:** 2024-03-28

**Authors:** Myoung-eun Park, Kevin K. Byon

**Affiliations:** ^1^Department of Physical Education, Korea National Sport University, Seoul, Republic of Korea; ^2^Department of Kinesiology, Indiana University, Bloomington, IN, United States

**Keywords:** super-leadership, self-leadership, exercise commitment, university Taekwondo team, structural equation modeling

## Abstract

**Introduction:**

Promoting super-leadership is crucial for the sustainable growth of college sport teams, especially as teams are experiencing a noticeable shift towards a more horizontal dynamic, where athletes themselves are emerging as leaders. However, there is a lack of research on the effectiveness of super-leadership and its possible outcomes in the context of collegiate Taekwondo teams.

**Methods:**

This study aims to investigate the impact of super-leadership on athletes’ self-leadership and exercise commitment and examine the mediating role of self-leadership in this relationship among collegiate Taekwondo athletes in South Korea. A total of 147 survey data were analyzed by structural equation modeling.

**Results:**

The findings revealed that super-leadership was found to have a positive impact on both athletes’ self-leadership (*β* = 0.71, *p* < 0.001) and exercise commitment (*β* = 0.30, *p* < 0.05). Additionally, the study reveals athletes’ self-leadership significantly impacts exercise commitment (*β* = 0.34, *p* < 0.05). Our findings also demonstrate that self-leadership was identified as a partial mediator in the relationship between super-leadership and exercise commitment (∆*χ*^2^ = 4.46, *p* > 0.05).

**Discussion:**

Theoretical and practical implications were discussed based on the current study’s findings.

## 1 Introduction

Collegiate sport teams invest significant resources and effort in talent development and performance enhancement. This investment not only enhances the team’s competitiveness but also forms a cornerstone for sustainable organizational development (Park et al., 2013). The intricate relationship between players and coaches assumes a pivotal role in nurturing the ongoing growth of collegiate sport organizations. Coaches extend beyond their roles as on-field mentors, exerting substantial influence over athletes’ psychological well-being, personal growth, and athletic abilities. This profound connection and the guidance provided by coaches are integral to the holistic development of athletes, ultimately contributing to the overall success of the organization. As a result, the effective leadership exercised by coaches exerts a considerable influence on athletes’ performance and personal evolution, further bolstering the organization’s growth (Kim and Kim, 2000). However, effective leadership faces challenges in this context due to increasing demands on coaches and the complex balance between individual player needs and team goals (Jeong and Hong, 2019). Despite these challenges, effective leadership, especially super-leadership, which empowers organization members to demonstrate independent leadership while nurturing future leaders, is increasingly recognized as a crucial leadership style for enhancing collegiate sport teams’ sustained growth and development (Manz and Sims, 1980; Manz and Sims, 2001; Jung and Kim, 2010; Kim and Yun, 2011; Kim, 2020).

Traditional sport leadership theories, such as transformational and servant leadership, emphasize a hierarchical relationship between leaders and athletes (Choi, 2011). However, there is a noticeable shift toward a more horizontal dynamic, where athletes within the team are developing into leaders. In this evolving landscape, super-leadership emerges as a relevant theory for sport settings (Kim, 2020). Super-leadership promotes self-leadership among athletes by guiding them to develop and take ownership of their capabilities and skills. It involves leaders partially delegating authority, granting athletes autonomy to explore ways to improve their motor skills, and instilling a sense of ownership in their training. As a result, leaders’ super-leadership is closely linked to self-leadership, emphasizing autonomy and spontaneity among team members, and further research is needed to explore this relationship specifically within the college sport domain, building upon studies conducted in general organizational settings (Jung and Kim, 2010; Kang et al., 2012; Park, 2012; Kim et al., 2016; Lee et al., 2016; Seok and Joo, 2016; Han and Cho, 2021a).

For athletes, self-management is essential to successful athletic performance, goal-oriented physical and mental training, and personal daily life. Athletes who lack self-management skills can fall behind in the competitive world of athletics, resulting in diminished performance and career loss (Kim, 2023). Interestingly, self-leadership is an effective self-management strategy for athletes (Park and Nam, 2017). It enhances their ability to evaluate and adjust themselves through self-reflection and self-management during task performance (Ahn and Nam, 2013). Additionally, self-leadership empowers athletes to control and regulate their emotions when facing challenging obstacles (Jun, 2013). Given that, athletes’ self-leadership has a positive effect on overall performance, such as exercise commitment (Song and Kim, 2013; Hong and Lee, 2022) and confidence in athletic abilities (Eun and Lim, 2014; Kim, 2016) and sport performance (Baek and Jang, 2017; Lee and Kim, 2017; Jung et al., 2020). Therefore, self-leadership is important in forming sport confidence and commitment and is considered an effective self-management strategy to improve overall athletic performance.

Meanwhile, exercise commitment is associated with complete engagement and identification with sport activities during training and competitions, in which individuals experience pleasure and intrinsic motivation without being influenced by external factors (Jung et al., 2020). Concurrently, the experience of being committed to sport plays a vital role in motivating individuals to persist in their athletic pursuits, as it brings pleasure and satisfaction (Kim and Yun, 2011). Additionally, leadership behaviors significantly influence athletes’ commitment to exercise (Hong and Lee, 2022). Ultimately, attaining a high level of exercise commitment is crucial in determining an individual athlete’s performance, underscoring the importance of complete commitment to exercise for optimal results (Song, 2014). Regrettably, the literature on the relationship between super-leadership and sport commitment within the context of college sport teams is notably limited. However, studies conducted within general organizations in various industries consistently demonstrate a positive impact of super-leadership on organizational commitment, representing employees’ identification and dedication to an organization (Jung and Kim, 2010; Jun, 2013; Han and Cho, 2017). Consequently, there is a significant research gap in investigating the influence of leaders’ super-leadership on exercise commitment in collegiate sport teams, emphasizing the need for further investigation.

Given the transition of the athlete-leader relationship from vertical to horizontal in collegiate sport teams, emphasizing player autonomy and sustainable team growth, it is appropriate to explore super-leadership as a key factor in acquiring competitiveness and developing effective leadership strategies. Additionally, within an organization, the effectiveness of super-leadership in fostering employees’ self-leadership and achieving positive behavioral outcomes varies depending on factors such as members’ tasks and organizational characteristics (Kim et al., 2011). However, there is a lack of research on the effectiveness of super-leadership and its possible outcomes in the context of collegiate Taekwondo teams (Jung and Kim, 2010; Choi, 2011; Kim and Yun, 2011; Kim, 2020). Therefore, this study aims to investigate the impact of super-leadership on athletes’ self-leadership and exercise commitment and examine the mediating role of self-leadership in the relationship between super-leadership and exercise commitment among collegiate Taekwondo athletes in South Korea.

More specifically, this study aims to investigate: (1) the relationships among super-leadership of team leaders, self-leadership of athletes, and sport commitment, (2) the relationship between self-leadership and sport commitment, (3) whether self-leadership plays a partial or full mediating role in the relationship between super-leadership and sport commitment among collegiate sport players. Understanding the dynamics between super-leadership, self-leadership, and sport commitment will enable coaches and managers to optimize their leadership approaches and create an environment that fosters motivation and success among collegiate Taekwondo athletes.

## 2 Literature review and hypothesis development

### 2.1 Relationship between super-leadership and self-leadership

Super leadership, initially introduced by Manz and Sims (1989), refers to a leadership style that aims to unleash the potential and maximize the efforts of oneself and subordinates. It involves guiding and empowering subordinates to become self-leaders and take responsibility for their own leadership. Super leadership entails a leadership approach where the leader shares the vision and information with team members, delegates authority, inspires their morale, values their autonomy, and creates conditions for them to exercise their creativity, ultimately maximizing overall work performance (Houghton and Yoho, 2005). Under the influence of super leadership, self-leadership allows individuals to proactively change themselves, set standards, understand their roles and responsibilities within an organization or team, comprehend the purpose behind their actions, and determine how to carry them out. It is a behavioral and cognitive strategy employed to influence oneself (Manz and Sims, 1980).

Self-leadership is a dynamic and empowering process that enables individuals within an organization to proactively take on leadership roles. It involves the intentional use of both behavioral and cognitive strategies by organizational members to exercise self-influence over their actions and decision-making (Manz and Sims, 1991). To be more explicit, self-leadership necessitates the demonstration of autonomous and responsible behaviors and attitudes, which play a pivotal role in achieving the organization’s goals and objectives (Ha, 2022). According to Manz (1986), self-leadership encompasses an action-oriented strategy that suppresses undesirable behaviors and promotes successful performance through positive actions. It also involves a natural reward strategy linked to finding enjoyment in tasks and a constructive thought pattern strategy that visualizes successful performance. Furthermore, super-leadership nurtures self-leadership in members through advanced job training (Jung and Kim, 2010), enabling them to achieve their own goals (Jeong and Choi, 2015) and promoting a stable psychological state. This spreads an organizational atmosphere conducive to effective goal achievement (Manz and Sims, 2001). Thus, organizational members who effectively employ self-leadership strategies exhibit higher levels of performance, strong task commitment, and the ability to solve key problems accurately.

The theoretical framework between super-leadership and self-leadership can be characterized as empowering and mutually reinforcing. Super leadership, which is based on McGregor’s Theory Y, operates on the belief that individuals have a natural inclination toward positivity and proactivity (Manz and Sims, 1989). It posits that when leaders embrace this perspective and grant autonomy to organizational members while effectively managing the organization, employees respond by actively and creatively engaging in their work. They assume the role of self-leaders who take control of their own development and progress. This theory highlights that super leadership, in contrast to traditional authoritative and hierarchical leadership, shifts the focus from top-down, leader-centered control to a model that encourages employees to reinforce and enhance their self-leadership. It achieves this by promoting self-assessment, self-reflection, and self-goal setting among employees. In this way, super leadership serves as a catalyst for stimulating and promoting self-leadership latent within employees. It empowers individuals to be self-motivated and self-driven, ultimately fostering a positive, proactive, and self-improvement-oriented culture within the organization.

Prior studies have demonstrated the significant impact of leaders’ super-leadership on enhancing members’ self-leadership in organizational contexts. Han and Cho (2021b) discovered a significant influence of super-leadership among church cell leaders on members’ self-leadership, with the effect being moderated by levels of mobile messenger communication between leaders and members. Kang et al. (2012) found a positive association between leaders’ super-leadership and employees’ self-leadership in six-star hotels, with self-leadership as a significant mediator in the relationship between super-leadership and employees’ creativity. Park (2012) also demonstrated the effect of managerial super-leadership on nurses’ self-leadership, concluding that higher employee motivation from leaders positively impacted nurses’ self-leadership. Consequently, the existing research highlights the significant role of super-leadership in enhancing self-leadership within organizational settings. Building upon previous research, this study advances the following hypothesis:

*Hypothesis 1 (H1)*: Leaders’ super-leadership will have a positive impact on self-leadership among college Taekwondo athletes.

### 2.2 Relationship between super-leadership and exercise commitment

Commitment to exercise is one of the key factors in improving athletes’ performance. Athletes who are highly engaged in a specific sport prioritize their involvement in sport, experience increased satisfaction, and demonstrate a psychological drive to enhance their competence (Kim and Yun, 2011; Kim et al., 2015). Furthermore, exercise commitment generates an optimal psychological state for sport performance and serves as internal motivation for players to fully engage in exercise, enhancing performance through focused concentration of both body and mind (Kim and Jung, 2018). To date, there seems to be no research establishing a direct link between leaders’ super-leadership and exercise commitment in collegiate sport. However, within the broader organizational context, the existing body of research has been conducted to explore the relationship between leaders’ super-leadership and organizational commitment, reflecting the identification and dedication of an employee toward their organization (Jung and Kim, 2010; Han and Cho, 2017; Park and Nam, 2017). For example, Han and Cho (2017) found a positive influence of super-leadership, among various leadership approaches, on organizational commitment among daycare school teachers. They concluded that super-leadership was the most influential factor for organizational commitment and effectiveness. Jun (2013) further established that super-leadership significantly positively impacted organizational commitment, job satisfaction, and job performance, emphasizing that employees with a strong organizational commitment are more likely to achieve organizational goals through their high work competence and job satisfaction. Based on the prior studies, the following hypothesis can be suggested:

*Hypothesis 2 (H2)*: Leaders’ super-leadership will have a positive impact on exercise commitment among college Taekwondo athletes.

### 2.3 Relationship between self-leadership and exercise commitment

Self-leadership is a cognitive and behavioral strategy used by individuals to exert influence on themselves, while super-leadership focuses on managing the entire organization. Self-leadership reflects a form of followership, where individuals willingly follow their actions to lead themselves (Manz and Sims, 2001). Self-leaders foster self-direction and self-motivation and take ownership of their actions to effectively lead themselves (Neck et al., 1995). More importantly, self-leaders exhibit emotional responses, such as a strong sense of unity and attachment to their organization, due to heightened psychological ownership during task performance (Neck and Houghton, 2006). Similarly, it is plausible that college athletes who adopt self-leadership are more likely to experience satisfaction with their training activities and team environment, demonstrating a strong commitment to their exercise. In fact, a substantial body of research in the sport context demonstrates a positive relationship between self-leadership and exercise commitment. Jung et al. (2020) reported that self-leadership among Taekwondo athletes significantly impacted exercise commitment and perceived performance and concluded that self-leadership serves as an effective self-management strategy, fostering positive emotions and enhancing overall athletic performance. Song and Kim (2013) also demonstrated that self-leadership had a positive effect on exercise commitment, eventually enhancing team spirit among volleyball players. Based on previous research, these results underscore the significance of self-leadership as a determinant of athletes’ commitment and performance. In light of these findings, we put forth the following hypothesis:

*Hypothesis 3 (H3)*: Self-leadership will have a positive impact on exercise commitment among college Taekwondo athletes.

### 2.4 Mediating effect of self-leadership on the relationship between super-leadershp and exercise commitment

Super-leadership and self-leadership directly impact subordinates’ organizational commitment (Manz and Sims, 2001). Super-leadership, which emphasizes employee autonomy and empowerment, provides clear direction and guidance, allowing the organization to progress effectively. Simultaneously, subordinates receive comprehensive job training, enhancing their understanding of their roles and task performance. These processes foster subordinates as self-leaders (Neck and Houghton, 2006). Furthermore, leadership facilitating factors, such as employees’ self-leadership, complement specific leader behaviors like leaders’ super-leadership. These external elements magnify the impact of leadership practices, playing a crucial role alongside the leader’s actions. They enhance subordinates’ organizational performance, job satisfaction, and organizational commitment (Manz and Sims, 2001).

**Figure 1:**
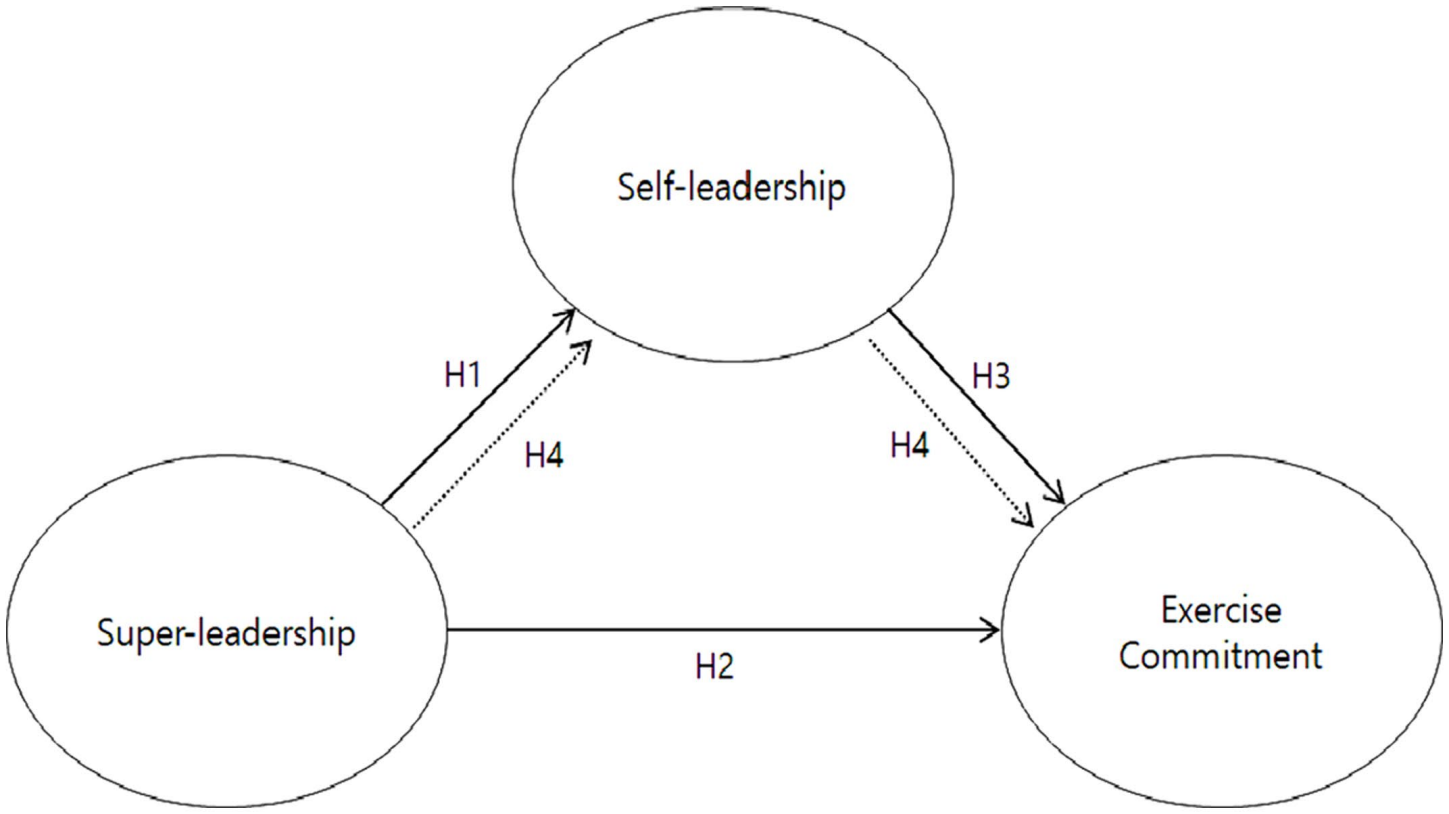
Research model.

In light of this, Lee et al. (2016) found that the perception of professors’ super-leadership among college students influences their self-leadership, which subsequently impacts learning flow and major satisfaction, fully mediating the relationships between super-leadership and learning flow. In contrast, Kim et al. (2016) reported that in college students majoring in physical education, professors’ super-leadership directly affects students’ self-leadership and learning immersion, while also indirectly impacting learning immersion through self-leadership, suggesting a partial mediating role of self-leadership in this relationship. Similarly, in a study involving employees from diverse industries, Kim et al. (2011) identified a partial mediating effect, demonstrating that self-leadership is a mediator in the relationship between super-leadership and organizational commitment. Considering the findings presented, the extent to which self-leadership acts as a full or partial mediator in the examined relationships remains uncertain. Hence, it is imperative to undertake additional research to investigate this matter and enrich our understanding of super-leadership. This study proposes the following hypothesis and presents a research model in Figure 1.

*Hypothesis 4 (H4)*: Self-leadership will have a partial mediating role in the relationship between leaders’ super-leadership and exercise commitment among college Taekwondo athletes.

## 3 Methods

### 3.1 Participants and data collection procedure

In order to achieve the goals of this study, a survey methodology was employed to select Taekwondo athletes from four universities in South Korea, specifically located in the Seoul, Gyeonggi, and Busan regions. The data collection took place in May 2023. Before data collection, the researchers communicated with the coaches and managers of the Taekwondo teams from the participating universities, seeking their collaboration and engagement in the study. Through telephone discussions, the researcher comprehensively explained the study’s objectives and the data collection process. Once the participation of the university Taekwondo teams was confirmed, the researchers personally visited each team. Prior to their training sessions, a meeting was conducted to present the study’s purpose and address any concerns regarding voluntary participation among the athletes. Written consent was obtained from all participants who willingly agreed to participate in the study.

For data collection, a self-administered survey method was utilized in this study. The participants were given questionnaires and allowed to complete them independently. The researchers collected the completed questionnaires on-site promptly to ensure data integrity. Initially, a total of 150 survey questionnaires were collected. After eliminating three duplicate or incomplete responses, the final sample size for statistical analysis comprised 147 participants.

Table 1 shows an overview of the demographic characteristics of the study participants. Among the total sample size, 94 were identified as male, while 53 were female. In terms of academic year, the study encompassed 4 first-grade students, 99 s-grade students, 32 third-grade students, and 12 fourth-grade students. A majority of the participants (71.4%) reported having a Taekwondo training experience ranging from 5 to 7 years. Additionally, it is noteworthy that all participants in the study were engaged in Kyorugi, a free-style sparring competition within Taekwondo.

**Table 1:** Demorgraphics of participants, descriptive statistics and correlations of variables.

Variables	Categories	Frequenty(%)	Factors	*M*	*SD*	Skewness	Kurtosis	1	2	3
Gender	Male Female	94 (63.9) 53 (36.1)	1 Super-leadership	4.25	0.50	−1.28	1.89	1		
Grade	1stt grade 2nd grade 3rd grade 4th grade	4 (2.7) 99 (67.3) 32 (21.8) 12 (8.2)	2 Self-leadership	4.10	0.59	−1.78	1.83	0.60**	1	
Training Length	Less than 5 years 5–7 years More than 7 years	12 (8.2) 105 (71.4) 30 (20.4)	3.Exercise commitment	3.96	0.50	−1.05	1.79	0.43**	0.43**	1

M, mean; SD, standard deviation. **p < 0.01.

Taekwondo athletes in this study undergo rigorous training twice daily, morning and afternoon, for five days each week. Morning sessions focus on 90 min of aerobic exercises such as running and interval training, alongside activities to enhance muscular endurance and agility like sit-ups, lunges, and burpee tests. Afternoons involve approximately 2 h and 30 min of stretching, fundamental strength exercises, Taekwondo techniques, and tactical training. Technical drills include kicks and combinations, while tactical sessions simulate specific match scenarios emphasizing kicking and connecting kicks in a virtual environment.

### 3.2 Instruments

In this study, data were collected using a survey consisting of 18 items, which included four demographic characteristics: gender, grade, length of training, and type of competition. The survey items were adapted from previously validated studies. Six items were adapted from the super-leadership scale, which originally consisted of 7 items with an alpha coefficient of 0.909, used in the research conducted by Lee et al. (2016) to assess super-leadership. In the course of an expert meeting that involved three sport psychology and management professors and two general managers from the university taekwondo teams, one of the original seven items, “My coach allows me to independently consider the technique before I start,” was omitted due to its redundancy with other survey questions.

Additionally, four items were adapted from the self-leadership scale, which comprised eight items and was utilized by Choi (2011). Initially, all eight items from Choi’s self-leadership scale were adapted. However, subsequent to conducting a primary confirmatory factor analysis, it was observed that the standardized coefficient values for four self-leadership items fell below 0.5. Consequently, the analysis excluded four specific items: ‘I do not perceive challenges during exercise as hindrances but as opportunities,’ ‘I excel in my work,’ ‘I establish my own objectives,’ and ‘When confronted with a challenging problem, I approach it with a positive mindset for resolution rather than negativity.’

Four items were adapted from the exercise commitment scale of Yoo (2019), which consisted of five items with an alpha coefficient of 0.942, to assess exercise commitment. During the expert meeting, one of the original five items, specifically, “I can control my learning during a training session,” was excluded due to its redundancy with other survey questions.

All items, except for the demographic characteristics, were rated on a 5-point Likert-type scale, ranging from 1 (strongly disagree) to 5 (strongly agree). To ensure content validity, a consensus meeting was held with a panel of three sport psychology and management professors and two general managers from the university taekwondo teams. The purpose of the meeting was to thoroughly review and evaluate the questionnaire items, with a specific focus on aspects such as language, sentence clarity, and potential word ambiguity. Furthermore, confirmatory factor analysis (CFA) and reliability tests were conducted to validate and assess the reliability of the questionnaire.

### 3.3 Data analysis

The statistical analyses conducted in this study comprised descriptive analysis, reliability analysis, confirmatory factor analysis (CFA), and structural equation modeling (SEM). SPSS version 23.0 and AMOS 23.0 software were employed for the statistical analyses. Descriptive analysis was performed to summarize and describe the data, providing information on the central tendency and variability of the variables. Reliability analysis was conducted to assess the internal consistency and reliability of the measurement items. CFA was utilized to evaluate the psychometric properties of the measurement model. The model fit, convergent validity, discriminant validity, and reliability were assessed using the maximum likelihood estimation procedure. Multiple indices, including chi-square, CFI (>0.90), IFI (>0.90), TLI (>0.90), RMSEA (<0.08), and SRMR (<0.08), were employed to evaluate the overall model fit (Hair et al., 1998). Convergent validity was assessed through the average variance extracted (AVE) values, which indicate the amount of variance captured by the latent construct. Discriminant validity was assessed by comparing the AVE values with the squared correlation between constructs. Finally, SEM was utilized to test the research hypotheses, allowing for the examination of relationships between variables and validation of the proposed theoretical model.

## 4 Results

### 4.1 Descriptive statistics

The descriptive statistical analyses indicated that the skewness values ranged from −1.78 to −1.05, and the kurtosis values ranged from 1.79 to 1.89, all falling within acceptable ranges (Hair Jr. et al., 1998). To examine multicollinearity, tolerance (0.63) and variance inflation factor (1.58) values were assessed, indicating that multicollinearity was not a concern (Fornell and Larcker, 1981). Descriptive statistics and correlations are presented in Table 1.

### 4.2 Measurement model test

The results of the measurement model evaluation are presented in Table 2. The measurement model assesses the reliability and validity of the indicators used for the constructs in the research model by examining their convergent and discriminant validity. The findings indicate that the measurement model demonstrated acceptable fit indices, with CFI of 0.92, IFI of 0.92, TLI of 0.90, SRMR of 0.05, and RMSEA of 0.07. The composite reliability (CR) values ranged from 0.86 to 0.92, and the average variance extracted (AVE) values ranged from 0.73 to 0.80, indicating good convergent validity (Hair Jr. et al., 1998). Furthermore, all AVE values exceeded the squared correlation of all pairs, confirming discriminant validity (Fornell and Larcker, 1981).

**Table 2:** Results of the measurement model test.

Items		λ	CR	AVE	α
	My coach encourages athletes to thoroughly review training before conducting it.	0.62			
	My coach encourages athletes to set their own training goals.	0.73			
	My coach advises athletes to solve problems independently during training.	0.66			
Super leadership	My coach praises and encourages athletes when they perform well during training.	0.83	0.92	0.66	0.80
	My coach inspires athletes to have the confidence to enhance their performance during training.	0.83			
	My coach allows athletes to recognize and acknowledge their achievements.	0.65			
	When I encounter a problem, I try to solve it on my own.	0.69			
	I strive to participate in additional activities besides the training given to me.	0.71			
Self-leadership	I reflect on the strategies I will employ to achieve my training goals.	0.67	0.86	0.61	0.73
	I reflect on the progress of my athletic performance.	0.68			
	I become so immersed in training that I lose track of time.	0.82			
	I believe that a training session is an opportunity to create valuable experiences.	0.84			
Exercise commitment	During training, I remain unaffected by external distractions.	0.58	0.90	0.72	0.78
	I tend to maintain a high level of focus during training sessions.	0.53			

*χ*^2^ = 134.06 (df = 74, *p* = 0.001), CFI = 0.92, IFI = 0.92, TLI = 0.90, SRMR = 0.05, RMSEA = 0.07. CR = composite reliability, AVE = average variance extracted, α = Cronbach’s alpha.

### 4.3 Common method variance

To confirm the absence of common method variance (CMV) bias in the results of our current study, we conducted a thorough examination. CMV is defined as “variance attributed to the measurement method rather than the constructs the measures represent” (Podsakoff et al., 2003). This investigation was vital because the data were collected from the same source (e.g., Taekwondo practitioners), and both predictor and outcome variables were measured concurrently. To address potential CMV bias, we employed Harman’s single factor method (Andersson and Bateman, 1997). In this method, we loaded all the variables used in our model into an exploratory factor analysis (EFA) using principal component analysis (PCA) with an unrotated factor solution. The results revealed that a single factor explained 35.33% of the total variance. Scholars generally suggest that if the total explained variance is less than 50%, CMV is not a significant concern (Andersson and Bateman, 1997).

### 4.4 Structural equation modeling

The results of the structural equation modeling (SEM), as presented in Table 3, indicated a satisfactory fit of the model to the data (*χ*^2^ = 134.06, df = 74, *p* = 0.001, CFI = 0.92, IFI = 0.92, TLI = 0.90, RMSEA = 0.07, and SRMR = 0.05). The SEM analysis revealed that leaders’ super-leadership had a significant positive impact on self-leadership (β = 0.71, *p* < 0.001) and exercise commitment (β = 0.30, *p* < 0.05). Self-leadership also had a significant positive effect on exercise commitment (β = 0.34, p < 0.05). Therefore, hypotheses 1, 2, and 3 were supported. Additionally, there were no significant confounding effects observed with regard to gender, grade, and the duration of training in the relationships between super-leadership, self-leadership, and exercise commitment.

**Table 3:** Results of hypothesis testing.

*Path*		*β*	*S.E*	*p*
H1	Super-leadership → Self-leadership	0.71	0.20	0.001
H2	Super-leadership → Exercise commitment	0.30	0.20	0.05
H3	Self-leadership → Exercise commitment	0.34	0.12	0.05
Confounding effects of demographic variables	Gender	−0.05	0.10	0.55
	Grade	−0.06	0.06	0.36
	Length of training	−0.01	0.08	0.89

### 4.5 Mediating effects of self-leadership

The present study examined the mediating effects of self-leadership on the relationship between super-leadership and exercise commitment by comparing two competing models: partial mediation and full mediation. Table 4 presents the results, demonstrating no significant differences in the model fit measures between the partial and full mediation models.

**Table 4:** Model fit measures and latent path coefficients for two models.

	*χ* ^2^	df	CFI	TLI	IFI	RMSEA	SL → EC	SL → SFL	SFL → EC
							*β*	*β*	*β*
Partial mediation model	134.06	74	0.92	0.90	0.92	0.07	0.30*	0.71***	0.34*
Full mediation model	138.55	75	0.92	0.90	0.92	0.07		0.74***	0.61***

SL, super-leadership; SFL, self-leadership; EC, exercise commitment. *p < 0.05, ***p < 0.001.

Specifically, the chi-square value increased by 4.49 in the full mediation model compared to the partial mediation model, and this difference was not statistically significant with 1 degree of freedom at a significance level of 0.05. The values of CFI, TLI, IFI, and RMSEA did not differ between the two models.

Furthermore, the path coefficients for both rival models were found to be statistically significant, with no notable disparities in the magnitude of the path coefficient values, except for the path from self-leadership to exercise commitment. Consequently, hypothesis 4 was supported, which proposes the partial mediating role of self-leadership in the relationship between super-leadership and exercise commitment.

## 5 Discussion

### 5.1 Theoretical implications

This study aimed to investigate the impact of super-leadership on athletes’ self-leadership and exercise commitment and examine the mediating role of self-leadership in this relationship among collegiate Taekwondo athletes in South Korea. As shown in Table 3, the current study’s results demonstrate that super-leadership significantly impacts Taekwondo athletes’ self-leadership as supported by prior studies (Kang et al., 2012; Park, 2012; Seok and Joo, 2016; Han and Cho, 2021b). Han and Cho (2021b) reported a positive impact of church leaders’ self-leadership on members’ willingness to continue participating in group sessions and their self-leadership, aligning with the findings of this study. Similarly, Seok and Joo (2016) supported the results of this study by demonstrating that the perceived super leadership of professors among college students positively affects self-leadership sub-factors such as goal achievement, voluntary action, and constructive thinking.

The positive linkage between super-leadership and self-leadership is rooted in the conceptual foundations set by Manz and Sims, who introduced the term in the 1980s (Manz and Sims, 1989). Their groundbreaking work represented a significant departure from conventional hierarchical leadership models, as Manz and Sims advocated for a paradigm shift toward a more collaborative and empowering approach. At the heart of the super-leadership concept, as envisioned by Manz and Sims, is the idea of leaders cultivating an environment that encourages followers to take on leadership roles themselves. This approach gives rise to a dynamic network of leadership within an organization, underscoring the interconnectedness of leaders and followers within a framework that is mutually beneficial and empowering. In essence, super-leadership establishes a symbiotic relationship between leaders and followers, transcending the constraints of traditional organizational hierarchies (Jun, 2013). Instead of directing and controlling, super leaders ensure autonomy and authority for organization members to fully embrace their roles. They encourage members’ voluntary actions and maximize individual abilities by providing the necessary motivation to fulfill their work effectively. Consequently, organizational members who embrace self-leadership under the influence of these super leaders set their own goals, increase their motivation, and develop a strong sense of responsibility toward achieving those goals, enhancing their work performance (Kang and Park, 2008). Thus, it is plausible that when collegiate Taekwondo athletes in the current study become well aware of their leaders’ super-leadership approach, in which they are given autonomy and empowerment over their Taekwondo training and verbal rewards regarding their athletic performance, they are likely to adopt a strong sense of self-leadership. This sense of self-leadership fostered by super leaders possibly empowers athletes to take initiative, set personal goals, and take responsibility for their training and development.

The current study also found that leaders’ super-leadership positively impacted exercise commitment among collegiate Taekwondo athletes (Table 3). Jung and Kim (2010) supported the findings of the current study by reporting that leaders’ super-leadership has a positive impact on organizational commitment, highlighting that leaders’ super-leadership strengthens their members’ commitment toward the organizations by providing opportunities of self-setting goals, self-observation, self-redesign, and self-compensation. Park (2017) further supported the findings of this study by demonstrating that super-leadership among leaders in small and medium enterprises positively influences organizational commitment and business performance. The study emphasizes that, although traditional leadership remains significant, enhancing organizational performance in today’s horizontal organizational environment necessitates a super leadership approach that fosters a sense of ownership and commitment among organizational members. Thus, leaders’ super-leadership perceived by collegiate Taekwondo athletes in the current study may enhance their exercise commitment as they feel respected by their coaches through given opportunities of ownership over their athletic activities, the chance to observe their progress, and the possibility of self-reward for their achievements.

Moreover, as revealed in Table 3, the current study found that college Taekwondo athletes’ self-leadership positively influenced exercise commitment, aligned with the prior studies (Jung et al., 2020; Kang and Kim, 2020; Hong and Lee, 2022). For example, Kang and Kim (2020) discovered that self-leadership among shooting athletes has a positive influence on resilience, exercise commitment, and sport satisfaction and concluded that these athletes enhance their self-belief to achieve personal goals and take control of their training, leading to not only increased exercise commitment but also a heightened sense of satisfaction. Hong and Lee (2022) further revealed that self-leadership, encompassing action-oriented, nature-compensatory, and constructive thinking, positively impacted exercise commitment among Taekwondo athletes. The study emphasized the importance of autonomy and self-control within Taekwondo, emphasizing that athletes who embraced self-leadership were more likely to dedicate themselves to their training. Athletes need training assuming specific competition scenarios and self-management as they prepare for various competitive situations, especially in taekwondo sparring matches, as success depends on effectively handling other opponents’ unexpected moves and techniques (Jung et al., 2020). To deal well with the fast-changing dynamics of competition, Taekwondo athletes must actively formulate and execute training plans and objectives considering these challenging circumstances. Athletes with the belief and confidence to achieve the desired performance by pursuing their self-designed training plans and goals are more committed to training and sporting activities, enhancing their overall athletic performance (Hong and Lee, 2022). Thus, it is plausible that as collegiate Taekwondo athletes in the current study adopt self-leadership characterized as goal-directed behavior, self-initiative, and intrinsic motivation, they are more likely to commit to their training and sport activities.

In the realm of super-leadership, where self-leadership is identified as a pivotal self-management strategy, a plausible connection to Maslow’s Hierarchy of Needs emerges. The essence of the super-leadership theory suggests that individuals, as they advance in their self-management capacities, may align with the hierarchical progression of needs delineated by Maslow. Maslow’s hierarchy of needs theory outlines a pyramid with five fundamental and essential categories of needs in ascending order: physiological needs, safety needs, social needs related to affection and belonging, esteem needs, and self-actualization (Maslow, 1987). The hierarchy implies that higher-level needs become relevant and influential once the lower-level needs are adequately satisfied. Similarly, for Taekwondo athletes engaged in self-leadership, the particular stage of Maslow’s Hierarchy of Needs that is closely associated is the esteem stage. This stage encompasses the athlete’s need for self-esteem, confidence, achievement, and the recognition of their abilities. Taekwondo athletes immersed in self-leadership are inclined to pursue mastery in their sport, cultivate confidence in their abilities, and aspire to gain acknowledgment and respect from their peers and coaches. Upon the fulfillment of needs associated with the esteem stage, Taekwondo athletes may progress to the highest level in Maslow’s hierarchy, known as the self-actualization stage. In the realm of Taekwondo, self-actualization for athletes might involve realizing their full potential in the sport, striving for personal excellence, and attaining a deep sense of fulfillment in their athletic pursuits.

Lastly, the current study exhibited that self-leadership partially mediated the relationship between leaders’ super-leadership and exercise commitment among collegiate Taekwondo athletes (Table 4) and supported the relevant prior literature (Kang and Park, 2008; Kim et al., 2011, 2016). Kim et al. (2016) found that college professors’ super-leadership significantly positively influenced students’ self-leadership and learning commitment, with self-leadership playing a crucial mediating role in this relationship. Furthermore, Kang and Park (2008) also corroborated our findings, showing that elementary and junior-high school principals’ super-leadership directly impacted teachers’ self-leadership and indirectly affected their organizational commitment, mediated by self-leadership. The authors conclude that shifting from the method of one-sided control by school principals in managing the school organization to a horizontal relationship that promotes information sharing and autonomy for teachers not only positively affected teachers’ commitment to the organization but also increased teachers’ motivation and voluntary action in their work, ultimately strengthening organizational commitment. Therefore, the current study empirically demonstrated that both the perceived super leadership of collegiate Taekwondo leaders by athletes and the self-leadership exhibited by athletes directly and positively affect athletic commitment, and self-leadership among athletes plays a crucial role in promoting leaders’ super-leadership concerning athletes’ athletic commitment.

### 5.2 Practical implications

The current study’s findings suggest practical implications for college Taekwondo coaches and managers. Collegiate Taekwondo coaches and managers should prioritize developing super-leadership qualities in themselves and promoting self-leadership skills among Taekwondo athletes. By fostering a coaching approach that provides autonomy, information sharing, and a supportive team culture, coaches can positively influence athletes’ self-leadership and commitment to training and competition. Encouraging athletes to take ownership of their training, set meaningful goals, and demonstrate initiative can increase motivation and voluntary actions, ultimately strengthening their commitment to the sport. Coaches and managers should also recognize the mediating role of self-leadership in the relationship between their super leadership and athletes’ commitment. By acknowledging the interplay between super leadership and self-leadership, coaches can tailor their coaching strategies effectively and promote a more empowering and motivating training environment.

Recognizing the uniqueness of each athlete is crucial in maximizing their commitment and potential. College Taekwondo coaches and managers should adopt a personalized coaching approach that caters to individual needs, strengths, weaknesses, and goals. Coaches can create a more effective and engaging training environment by understanding athletes’ specific requirements and providing tailored guidance and support. For instance, if an athlete expresses a desire to improve flexibility for advanced kicks, the coach can integrate specific stretching routines into their training program. Ongoing assessments then monitor progress toward these individualized goals. Recognizing diverse learning preferences among athletes is crucial—some may thrive with visual demonstrations, while others prefer hands-on practice. Aligning coaching strategies with individual learning styles significantly amplifies instructional effectiveness. Additionally, coaches can collaborate with athletes to set personalized goals, exemplified by an athlete aspiring to improve flexibility. The coach integrates tailored stretching routines into the training plan, and regular assessments track progress toward these individualized objectives. This personalized approach can build stronger coach-athlete relationships, enhance communication, and instill a sense of trust and dedication among athletes. Additionally, coaches should continuously seek opportunities for professional development and stay informed about the latest sport psychology and coaching practices. Staying up-to-date with relevant research and insights can help coaches adapt their strategies to best support athletes’ exercise commitment and performance in the dynamic world of Taekwondo.

## 6 Conclusions and limitations

### 6.1 Conclusion

The present study’s findings align with previous research, providing valuable insights into the relationship between super-leadership, athletes’ self-leadership, and exercise commitment among university Taekwondo athletes. We observed that super leadership positively influences self-leadership, highlighting the significance of effective leadership in fostering athletes’ self-driven capabilities. Moreover, the positive effect of the super-leadership on exercise commitment reinforces the critical role of supportive and empowering leadership in enhancing athletes’ dedication to their sport.

Furthermore, the study confirms the importance of self-leadership in shaping exercise commitment, with athletes who demonstrate self-driven behaviors and intrinsic motivation showing higher levels of dedication to their training and competition. Importantly, our findings highlight the mediating role of athletes’ self-leadership in the relationship between super-leadership and exercise commitment, emphasizing the need for coaches and managers to cultivate self-leadership skills among athletes to further enhance their commitment and performance.

### 6.2 Limitations and suggestions for future studies

Despite providing valuable insights, this study has limitations that need to be acknowledged. Firstly, the research concentrated exclusively on university Taekwondo athletes in South Korea, which could potentially constrain the applicability of the findings to other sport or athlete populations across diverse regions and countries. To enhance the study’s external validity, future research should encompass a broader spectrum of athlete populations, regions, and countries. Secondly, the present study employed a one-dimensional self-leadership scale rather than a comprehensive multi-dimensional self-leadership scale that encompasses aspects like constructive thinking, spontaneous behavior, and goal achievement behavior. The use of a single-dimensional self-leadership scale in this research imposes certain inherent limitations, primarily involving the oversimplification of the multifaceted self-leadership concept. Therefore, it is advisable to approach the interpretation of the current study’s findings with caution. To deepen our understanding of the relationships between super-leadership and self-leadership, it would be valuable to replicate this study in future research, employing a multi-dimensional self-leadership scale to enrich and broaden our insights. Thirdly, the study investigated the direct and indirect effects of a leader’s super-leadership on exercise commitment among collegiate Taekwondo athletes. However, it did not explore potential moderating factors, such as competition levels and athletes’ self-management levels, which could influence the relationship between leader’s super-leadership and athletes’ exercise commitment. This limitation may have hindered a comprehensive understanding of the relationship. Hence, future research should replicate and expand upon this study, considering key mediating factors that may strengthen the connection between super-leadership and exercise commitment among athletes. Lastly, the study relied on a survey methodology, potentially introducing limitations related to self-reporting and response biases. To improve validity, future research can adopt mixed-method approaches, incorporating observations, interviews, and surveys for a more comprehensive understanding of the subject.

## Data availability statement

The raw data supporting the conclusions of this article will be made available by the authors, without undue reservation.

## Ethics statement

Ethical approval was not required for the study involving humans in accordance with the local legislation and institutional requirements. The studies were conducted in accordance with the local legislation and institutional requirements. The participants provided their written informed consent to participate in this study.

## Author contributions

MP: Formal analysis, Investigation, Methodology, Writing – original draft. KB: Conceptualization, Data curation, Formal analysis, Investigation, Methodology, Project administration, Supervision, Writing – review & editing.
